# Computational Study of the Curvature-Promoted Anchoring of Transition Metals for Water Splitting

**DOI:** 10.3390/nano11123173

**Published:** 2021-11-23

**Authors:** Weiwei Liu, Youchao Kong, Bo Wang, Xiaoshuang Li, Pengfei Liu, Alain R. Puente Santiago, Tianwei He

**Affiliations:** 1 Department of Physics and Electronic Engineering, Yancheng Teachers University, Yancheng 224002, China; liuww@yctu.edu.cn (W.L.); yb87816@connect.um.edu.mo (Y.K.); 2 School of Applied Physics and Materials, Wuyi University, Jiangmen 529020, China; wangbo312@mails.ucas.ac.cn; 3 Spallation Neutron Source Science Center, Institute of High Energy Physics, Chinese Academy of Sciences, Dongguan 523803, China; pfliu@ihep.ac.cn; 4 Department of Chemistry and Biochemistry, University of Texas at El Paso, 500 W. University Avenue, El Paso, TX 79968, USA; arpuentesan@utep.edu; 5 Fritz-Haber-Institute der Max-Planck-Gesellschaft, Faradayweg, 4-6, 14195 Berlin, Germany

**Keywords:** 2D materials, transition metal dichalcogenides, strain engineering, single atom catalyst, water splitting

## Abstract

Generating clean and sustainable hydrogen from water splitting processes represent a practical alternative to solve the energy crisis. Ultrathin two-dimensional materials exhibit attractive properties as catalysts for hydrogen production owing to their large surface-to-volume ratios and effective chemisorption sites. However, the catalytically inactive surfaces of the transition metal dichalcogenides (TMD) possess merely small areas of active chemical sites on the edge, thus decreasing their possibilities for practical applications. Here, we propose a new class of out-of-plane deformed TMD (cTMD) monolayer to anchor transition metal atoms for the activation of the inert surface. The calculated adsorption energy of metals (e.g., Pt) on curved MoS_2_ (cMoS_2_) can be greatly decreased by 72% via adding external compressions, compared to the basal plane. The enlarged diffusion barrier energy indicates that cMoS_2_ with an enhanced fixation of metals could be a potential candidate as a single atom catalyst (SAC). We made a well-rounded assessment of the hydrogen evolution reaction (HER) and the oxygen evolution reaction (OER), which are two key processes in water splitting. The optimized Gibbs free energy of 0.02 for HER and low overpotential of 0.40 V for OER can be achieved when the proper compression and supported metals are selected. Our computational results provide inspiration and guidance towards the experimental design of TMD-based SACs.

## 1. Introduction

Hydrogen gas is considered the most plausible alternative to solve the energy crisis due to its green products, high heat of combustion, and sustainable properties [1,2,3]. Generally, electrolytic water is believed to be a clean and simple way to generate hydrogen compared with many other methods. Proper catalysts are usually used to speed this process. The selection of traditional catalysts focuses on noble metals (like bulk Pt), for their high stability, chemical activity and selectivity, and even inter-atomic cooperation [4,5,6]. But the low rate of exposed active sites, high cost, and limited storage create obstacles for the widespread application of traditional catalysts.

Mono- or few-layer two-dimensional (2D) materials, with plenty of exposed chemisorption sites, easy, low-cost fabrication, and high conductivity show great potential as potential candidates to replace the traditional catalysts for water splitting [7,8,9,10]. Transition metal dichalcogenides have aroused attention among 2D materials due to their wide range of bandgaps, strong chemical stability, and sandwich structure X–M–X since the first reported HER with MoS_2_ in 2005 [11]. At that time, only TMD edges were believed to act as effective sites for catalytic processes, because the bond-free surface was inert. But the exposed sites on edges are much less than that on the surface. Hence, kinds of methods are proposed to activate the surface, such as introducing strain and defects, as well as tuning phase and heterostructure construction [12,13,14,15]. These proposed methods can promote the catalytic performance of 2D materials to some extent, but low efficiency on a per atom basis still needs to be tackled [11,16]. 

The atomic efficiency and low unsaturated active sites quickly caught the attention of the catalysis community since the concept of single atom catalysts (SACs) was launched in 2011 [16]. Dispersing or anchoring metal atoms/clusters on TMD thin layers greatly improved the catalytic ability of TMD-based SACs [17,18]. However, the widely adopted doping and adsorption for anchoring single atoms on graphene cannot work well in a TMD system. The formation energy of doping in MoS_2_ is generally over 1.5 eV, and adsorption energy is much larger than that of doping on its basal plane, due to the weak adsorption of the S atom layer [14,19,20,21,22,23,24]. For example, Deng et al. systemically studied the possible pathways towards Pt anchoring on the surface of MoS_2_, that is, the doping and adsorption of Pt atoms to promote electrolytic activity [24]. They proved that the formation energy (E_f_) of Pt doping is much lower than that of Pt adsorption on the MoS_2_ because it was hard to realize 3S-Pt structure in basal plane. That is why, until now, most efforts focus on the doping manner rather than adsorption (generally E_f_ > 2 eV in calculations) to promote the catalyst performance of TMD for water splitting. We also noticed that the successful realization of 3N- or 4N- Metal structure in graphene could promote the fixation of single atoms to facilitate HER, oxygen reduction reaction (ORR), and oxygen evolution reaction (OER) [25,26,27,28]. Therefore, it is necessary to figure out a suitable method to both promote the E_f_ and the fixation of single atoms on TMD thin layers. 

Hence, we proposed a unique structure of MoS_2_ monolayers to support transition metals with the combination of strain engineering and transition metals’ decoration. We chose noble metal Pt and non-noble metal Fe for the assessment in our work. To evaluate the performance of metals on cMoS_2_, the adsorption energy E_ads_ and diffusion barrier were calculated as a function of compressions. The great reduction (72% Max) of E_ads_ at large curvatures indicates the more possible formation of M@cMoS_2_ than that on basal plane. The single atom would experience a strong fixation as the compression increases, due to the enlarged diffusion barrier up to three-fold of basal plane. We also find that E_ads_ would be further decreased under charging the situation with 1e^−^ or 2e^−^. The binding energy (E_b_) of H-Fe@cMoS_2_ and H-Pt@cMoS_2_ is clearly affected by curvatures. The strongest binding was achieved a at curvature of 4% for Pt@cMoS_2_ and 16% for Fe@cMoS_2_. Correspondingly, the optimized Gibbs free energy values were −0.02 eV at 16% for Fe@cMoS_2_ and 0.03 eV at 4% for Pt@cMoS_2_. The HER performance could be further improved with charging one or two electrons. We also demonstrate that the large dissociation barrier of H_2_O in alkaline solution resulted in a slow reaction rate of HER of the M@cMoS_2_ in alkaline environment. At last, the OER performances of Pt@cMoS_2_ and Fe@cMoS_2_ were evaluated at different curvatures. The overpotential of 16%-Pt@cMoS_2_ could be comparable to basal one but has much lower thermal energy for O_2_ desorption. The promoted HER and OER of Pt or Fe anchored on MoS_2_ would be expand to other cTMDs and transition metals. The computational results pave the way towards the development of very efficient cTMD-based water splitting electrocatalysts.

## 2. Computational Methods 

All the optimization and energy calculations are carried out with density-functional theory (DFT) to investigate the effect of surface curvature on the catalytic performance of cMoS_2_. The Perdew-Burke-Eznerhof generalized gradient approximation (PBE-GGA) [29] is used for the analysis of exchange and correlation potential. The projector augmented wave (PAW) method in the Vienna ab initio simulation package (VASP) is employed in this work [30,31]. The integration of the first Brillouin zone is carried out with the Gama scheme of k point sampling. The used supercell is about 33 Å × 10 Å × 20 Å (6$\sqrt{3}$ × 3 × 1 unit cells). A vacuum region of ≥15 Å is applied along the z axis to avoid the interaction between adjacent interlayers. All the calculation uses 5 × 10^−6^ eV/atom as the total energy convergence condition and 0.04 eV/Å as the maximum force convergence criteria. All the parameters are carefully tested before further calculations. Spin-polarization is considered in our calculation and the cutoff energy is set to 500 eV. For the evaluation of the kinetic analysis, we adopt the climbing image nudged elastic band (CINEB) method to figure out the diffusion energy barrier of metal atoms on cMoS_2_ and water dissociation of H_2_O molecular. The model construction of curved MoS_2_ monolayers is similar to our previous study [32].

The adsorption energy of metals on cMoS_2_ is calculated as:

△E_abs_ = E (cMoS_2_ + M) − E(cMoS_2_) − E(M)

where, the E (cMoS_2_ + M), E(cMoS_2_) and E(M) represent the total energy of monolayer curved MoS_2_ with one absorbed metal atom, the pure curved MoS_2_ and the metal, respectively. Since the calculation of E(M) is not unified in current research, we prefer to use the calculation method, that is, the energy of bulk metal divided by the number of metal atoms. The smaller the adsorption energy, the more stable the TMs would be on the curved structures.

The binding energy of bond H-M is calculated:

△E_b_ = E(M@cMoS_2_) + 1/2E(H_2_) − E (M@cMoS_2_ + H)

where, E (M@cMoS_2_ + H) stands for the energy of one H atom absorbed on the metal site. E(M@cMoS_2_) is defined as the energy of one metal atom anchored at the center site of the crest of cMoS_2_. E(H_2_) describes the total energy of the H_2_ molecule in its gas phase. The large binding energy indicates the strong adsorption capacity of the active site for hydrogen.

The process of hydrogen evolution reaction is considered in acidic solution [2,3,33]. The first step is that a proton from the solution absorbs on the slab and becomes an intermediate adsorbed hydrogen atom H_ad_.

H^+^ + e^−^ → H_ad_

The adsorbed H atoms then combine into a hydrogen molecule.

2H_ad_ → H_2_

Therefore, it is a simple calculation of the Gibbs free energy in acidic solution through the computational hydrogen electrode (CHE) method [34],

△E_H =_ E(M@cMoS_2_ + H) − E(M@cMoS_2_) − 1/2E(H_2_)

△G_H_ = △E_H_ + △E_zpe_ − T△S_H_

where E(cMoS_2_ + H) and E(cMoS_2_) represent the total energies of cMoS_2_ with and without one adsorbed H, respectively. △E_zpe_ is the difference in zero-point energies between the adsorbed H and H in the gas phase of hydrogen molecules. △E_zpe_ − T△S_H_ is calculated to be 0.24 eV [34].

## 3. Results and Discussion

### 3.1. Anchoring-Activity of Metal on cMoS_2_

**Figure 1:**
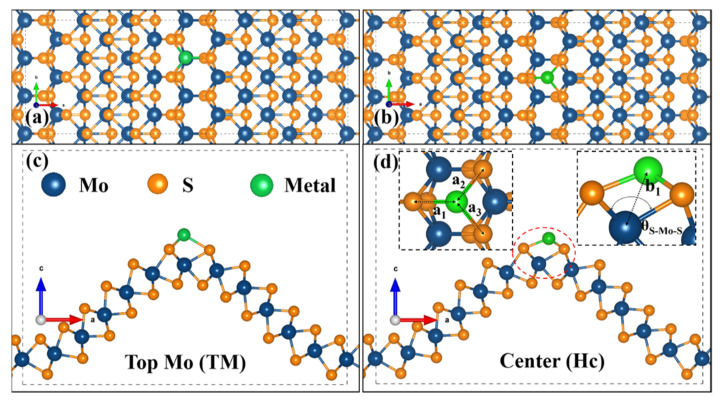
Representative configuration of the metal atom adsorbed on the crest of cMoS_2_. (**a**,**b**) are the top view of the TM site and Hc site; (**c**,**d**) are the corresponding side views.

Our previous study indicated that the curved MoS could promote the surface activation due to the low adsorption energy of H atoms, but the optimized Gibbs free energy of 1.25 eV is still high for practical applications [32]. The active surface of cMoS_2_ at large curvatures and the theoretically 100% efficacy of SACs catalysts could be combined to fabricate highly active catalysts. Hence, we chose noble metal (Pt) and non-noble metal (Fe) atoms to evaluate the possible adsorption of single atoms in our unique curved structures. Figure 1 shows the schematic diagram of the possible adsorption sites of metals at the crest of cMoS_2_, top Mo site (TM) and the center site (Hc) of honeycomb. The increasing curvature leads to a dramatic structure change along the armchair direction (Tables S1 and S2). For S-Fe (S-Pt), the a_1_ is changed from 2.10 Å (2.35 Å) to 2.59 Å (3.28 Å), but the a_2_ and a_3_ show a limited increase, from 2.010 Å (2.35 Å) to 2.27 Å (2.38 Å). Then, the enlarged space allows metal atoms to close the inner site of Mo, which can be proved by the decreasing distance (b_1_) of Fe-Mo (Pt-Mo). The b_1_ changes from 2.94 Å (3.54 Å) to 2.4 Å (2.76 Å). We also noticed that even though the θ_S-Mo-S_ are still increasing, the b_1_ keeps almost static after δ = 8%, which could be attributed to the repel effect between metal atoms.

**Figure 2:**
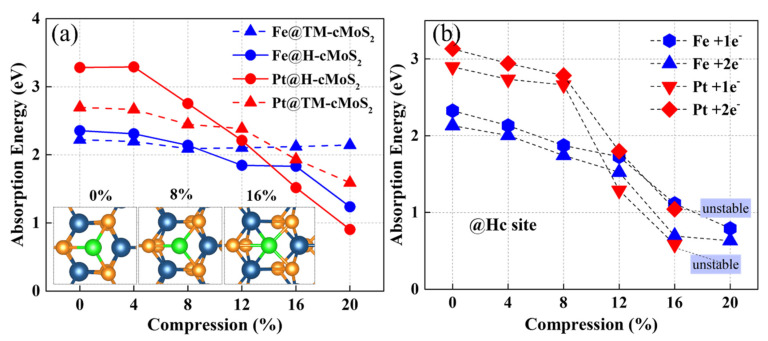
(**a**) The adsorption energy of metal atoms on neutral cMoS_2_ as a function of compressions. The insets represent the top view of M@cMoS_2_ at different compressions. (**b**) The adsorption energy of metal atoms on the H site of charged cMoS_2_ as a function of compressions.

The adsorption energy of metal on TM and Hc are clearly affected with the change of curvature (Figure 2). The E_abs_ of Pt on the basal plane (0%) of MoS_2_ is around 3.2 eV at Hc site and 2.6 eV at TM site, which is in agreement with previous studies [24]. As the compression increases, E_abs_ gradually reduces for Fe and Pt, but with different degrees. The Pt and Fe atoms prefer the Hc sites at larger curvatures (δ ≥ 12%) rather than the TM site due to the low calculated E_abs_ at Hc sites. When the compression gets to 20%, the E_abs_ at the Hc site could be much lower to 0.9 eV (1.2 eV) with nearly 72% (49%) decrement for Pt (Fe). Remarkably, we found that E_abs_ at TM sites show a divergence between Pt and Fe when δ ≥ 8%, that is, E_abs_ of Fe, keeps almost unchanged while Pt shows a continuous decrease. Our calculation found that Top S (tS) is the most impossible to adsorb metal due to the much larger adsorption energy than that of the other two sites, which is consistent with previous reports [24]. Besides, the curvature has no positive effect on the Fe or Pt adsorption activity at tS on cMoS_2_ (Figure S1).

The systems charged with one electron and two electrons were also calculated for simulating the real situation in experiments. Figure 2b shows that the trend of E_b_ at the H site is similar to the neutral situation. The increasing number of electrons into the system results in a promotion of the metal adsorption of cMoS_2_, that is, the E_ads_ with 1e^−^ is lower than that with 2e^−^. The difference of E_ads_ between 1e^−^ and 2e^−^ is enlarged at δ ≥ 12%, which would mean that the large curvature helps adsorbents attract more extra charge. The unstable structure of Pt-cMoS_2_ is observed when δ > 16%, which would result from the weak interaction of S-Pt induced by the enlarged a_1_ in a charged system (Tables S2–S4).

**Figure 3:**
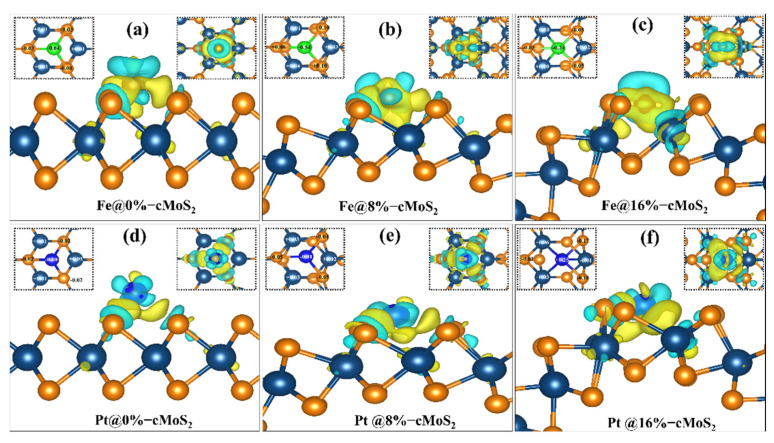
(**a**–**f**) The charge density difference between cMoS_2_ and Fe@cMoS_2_ (the upper)/Pt@cMoS_2_ (the lower). The yellow and blue represent the charge accumulation and dissociation. The left and right insets indicate the Bader charge change around absorbents and the top view of charge density difference for each situation. The isosurface value is set to 0.004 e/Å^3^.

To dig out the relationship between the adsorption energy and the curvature of cMoS_2_, the charge density difference isosurface and Bader charge transfer are calculated (Figure 3). In the sandwiched structure of MoS_2_, generally, the middle layer of Mo atoms is screened by outer S atoms, which makes the adsorbate hard to interact with the Mo layer. Moreover, the saturated surface bonds of the S atom layer show a weak adsorption of most adsorbates, leading to an inert chemical activity [35]. For the basal plane (0%) MoS_2_, the Pt and Fe atoms show less effect on the charge density of Mo (Figure 3a,d). As the curvature increases, the distance of Pt/Fe-Mo decreases (b_1_ in Tables S1 and S2). Then, a strong interaction between Pt/Fe and Mo appears at a large compression, which is indicated by the large charge density transfer (Figure 3c,f). The calculated Bader transfer also proves the electronic redistribution. For example, the Bader charges of Fe and Pt are −0.64 eV and −0.1 eV at 0%-cMoS_2_, which increase to −0.24 eV and +0.25 eV at 16%-cMoS_2_ (Table S3). The higher charge transfer indicates a stronger interaction. In addition, the charged electrons accumulate around the absorbates to further increase the ability of the adsorption. For example, the increasing electrons accumulate around Pt after more electrons charged into the system (Figure S2). Therefore, the more electrons accumulate around adsorbates, the stronger adsorption cMoS_2_ has. Because of the strong ability to adsorb single atoms, monolayer MoS_2_ with its curved deformation has the potential to act as a single-atom catalyst, which could be further proved by the AIMD simulation at room temperature (Figure S3).

**Figure 4:**
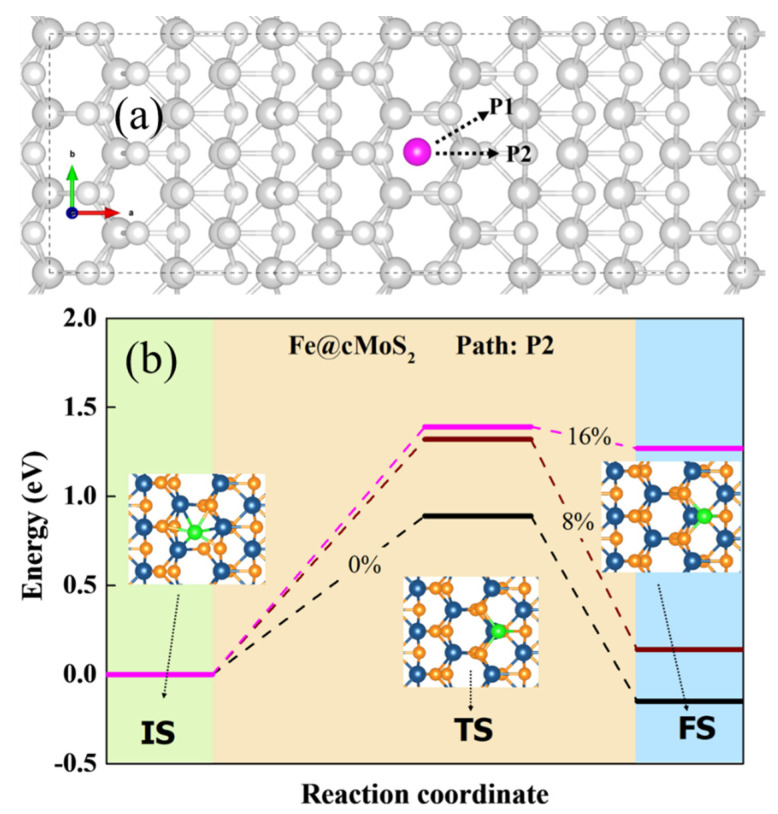
(**a**) The possible pathway of M diffusion in cMoS_2_. (**b**) The diffusion coordinates of the Fe atom in cMoS_2_ at different compressions. The inset is the corresponding top view of different states.

Another important issue of potential SACs focuses on their stability on the surface of the slab, meaning the single atom would not diffuse arbitrarily. Hence, we calculated the diffusion barrier of Pt and Fe on the crest of MoS_2._ Figure 4a shows the possible diffusion pathway for a single atom: P1 and P2. The results show that P2 is more possible than P1, because the E_b_ of top Mo along the P2 is lower than that of the bridge of S-Mo along P1. From Figure 4b, we can see the energy and optimized structures of three states when the Fe atom would diffuse on the surface of cMoS_2_. For a basal plane, the adsorption energy of Fe is slightly different (Figure 2a). The Fe atom needs to conquer an energy barrier of 0.89 eV to arrive to the TM site. The energy of finale state (FS) is a negative value, due to the lower E_ads_ at TM than that at Tc. But for Pt atoms, the large difference of E_ads_ between TM and Tc leads to a barrierless movement from Hc to TM on basal MoS_2_ (Figure S4).

As the curvature enlarges, Fe and Pt have difficulty going down the curved surface due to the increasing energy of the transition state (TS) (Figure 4b and Figure S4). In comparison with basal plane, the diffusion barrier experiences 1.6-fold (0.89 eV → 1.4 eV) and 3-fold (0.45 eV → 1.5 eV) increments for Fe and Pt, respectively. Additionally, atoms are difficult to anchor at other sites when δ ≥ 16%, since the energy of FS increases sharply from δ = 8% to δ = 16%, which would strengthen the fixation selectivity of metals adsorbed on cMoS_2_. Overall, the curvature-promoted single-atom-anchored on cMoS_2_ indicates a great potential as SACs.

### 3.2. Hydrogen Evolution Reaction

**Figure 5:**
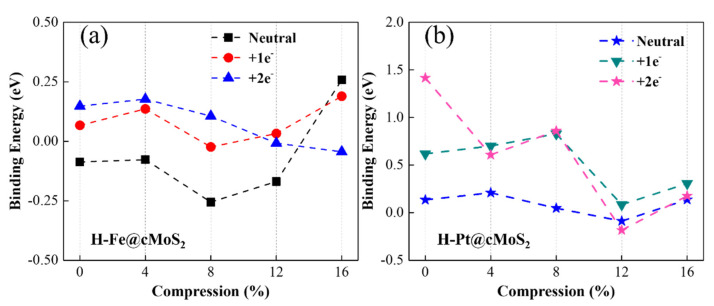
Binding energy of Fe@cMoS_2_ (**a**) and Pt@cMoS_2_ (**b**) as a function of compressions at neutral, +1e^−^ and 2e^−^ situations.

To evaluate the ability of water splitting based on metal-decorated cMoS_2_, we first investigated the hydrogen evolution reaction based on Fe@cMoS_2_ and Pt@cMoS_2_. The HER reaction mechanism depends on the pH of the solution. In acid solution, a sufficient H^+^ became indeterminates of H_ad_ after getting electrons from the electrode [2]. The key issue is that the surface of catalysts provides active sites to combine two adsorbed H_ad_ into H_2_ gas. Hence, we first calculate the binding energy (E_b_) of an H-M bond to evaluate the adsorption of H on Pt@cMoS_2_ and Fe@cMoS_2_. From Figure 5a,b, the basal plane of Pt@MoS_2_ is more active for the H adsorption than that of Fe@MoS_2_, due to the high binding energy 0.18 eV than 0.1 eV of Fe. The positive value (Figure 5b) indicates it is an exothermic process in a Pt-based catalyst, which is in agreement with other reports [36]. The E_b_ is fluctuating within the range of 0.5 eV along with the increment of the compression. The E_b_ of H-Pt initially goes up to 0.21 eV by adding small stress, and then linearly decreases to −0.08 eV (δ = 12%), indicating a weak trend of H adsorption. A dominant improvement of binding appears at large curvatures. H-Fe shows a similar trend, but the turning point (8%) is earlier than Pt, which is attributed to the magnetic influence. All in all, we find that the strongest binging of H-Pt appears at a small compression of 4%, while that of H-Fe is larger (δ = 16%). It should be noted that the structure of M@cMoS_2_ becomes unstable after one H atom absorbs on it under a large compression (>16%).

We then considered the charging situation. With one extra electron, the trend of binding energy is similar to the neutral one as compression increases (Figure 5a,b), except for the H-Pt@cMoS_2_ with 2e^−^ (Figure 5b). Because the extra electron leads to the shifting of the Pt adsorption position from Hc to TM and then induces a strange binding energy of H-Pt on 0%-cMoS_2_. Compared with a neutral situation, charging one electron brings a stronger binding between the H atom and the Fe/Pt atom at the small curvature (δ ≤ 8%), due to the much higher binding energy. For example, E_b_ of H-Fe is above zero with one extra electron, which means a spontaneous adsorption of the H atom. When the compression gets large, the binding energy is distinguished. The E_b_ with one electron is smaller than that in a neutral system for H-Fe after 16% and for H-Pt after 12%. This finding indicates the adsorption of H becomes weak, which would result in more 3d empty orbitals of metals being occupied by extra electrons, since the overlap of charge density between Mo and Fe or Pt gets larger at a high compression (Figure 3c,f). This conclusion can be further proved by the adsorption results with two electrons charging [37]. The curvature facilitates the injection of electrons to the adatoms (Fe, Pt) via the exposed Mo atom at a large compression, and then fewer empty orbitals lead to a weak adsorption.

**Figure 6:**
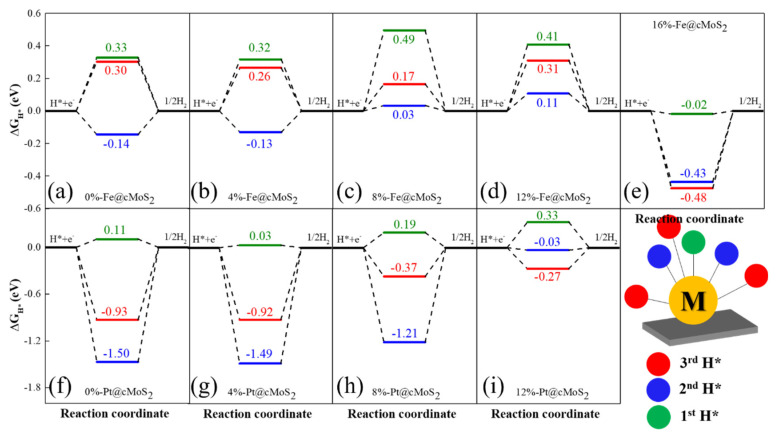
(**a**–**i**) The free energy diagram of Fe@cMoS_2_ and Pt@cMoS_2_ for hydrogen evolution reactions at different compressions. The Gibbs free energy of an ideal catalyst for the HER should be close to 0.

The Gibbs free energy (ΔG_H_) is widely used to judge the HER performance of a catalyst. Since HER involves two steps (H adsorption and H_2_ desorption), the optimized ΔG_H_ is equal to zero. For the specific site of unsaturated single atoms supported on cMoS_2_, we calculate the Gibbs free energy for the first (green), second (blue), and third hydrogen atoms (red) at different curvatures (Figure 6). For Fe@cMoS_2_ from 0% to 12%, we find that the high reaction activity appears when the second H atom is adsorbed, and the low HER activity with one H, due to the low reaction barrier at each compression (the blue line closes to 0 eV). The optimized ΔG_H_ of 0.03 eV is achieved at δ = 8%. But at large curvatures (16%), the high HER activity (ΔG = −0.02 eV) would be achieved when Fe@cMoS_2_ adsorbs one hydrogen, because the strong binding (ΔG = −0.43 eV) of H-Fe-H needs energy for H_2_ desorption with one more hydrogen adsorbed. Meanwhile, for Pt@cMoS_2_, the strong interaction between 2H/3H and Pt indicates the difficult desorption of H_2_ at small curvatures. Instead, the one electron is possible due to the lower reaction barrier. The optimized Gibbs free energy (0.03 eV) is located at 8%-Pt@cMoS_2_. But when compression gets to 12%, the Pt with two electrons owns high activity (ΔG = −0.03 eV). After 12%, Pt@cMoS_2_ shows an unstable structure with one more hydrogen absorbed. Therefore, the curved MoS_2_ with metals anchored have a great potential for HER under a proper compression force.

Additionally, the catalytic performance of Pt@MoS_2_ and Fe@MoS_2_ in neutral or alkaline solutions are also investigated. Different from the acid electrolyte, the proton is the minority in alkaline one. The first step is the water dissociation, in which the H_2_O molecule would adsorb on an active surface and then dissociate into OH^−^ and H^+^ [34]. The calculated dissociation barrier is larger than that of a traditional Pt/C electrode due to the weak binding between H and S atoms. All the details are discussed in the supporting information (Figures S5 and S6).

### 3.3. Oxygen Evolution Reaction

The oxygen evolution reaction (OER) is another key half reaction that involves four electrons transfer steps in the electrocatalytic water splitting, the same importance to HER. Therefore, a correct assessment of OER is beneficial for an in-depth understanding of the catalyst for water splitting. Up to now, the metal-based catalysts for OER mainly focus on the edge effect and edge-supported single atoms [38,39,40]. For instance, Xu et al. reported a Pt anchored at the edge of MoS_2_ demonstrated an ultra-low overpotential (η) of 0.46 V, which delivered a performance close to the bulk noble metal [38]. Similarly, the limited area of the edge restricted its massive application. The recent experimental results show that co-doping MoS_2_ exhibited a similar result (η_min_ = 0.48 V) to the edge MoS_2_ but provided sufficient active reaction sites [41]. This provides a clear direction to the TMD-based SACs. However, the large doping energy (>2 eV, in calculation) makes doping difficult to some extent [42,43]. The above discussed adsorption promotion of metals in our system would alleviate the problem to a more possible degree. Here, we calculate the four electron transfer steps and make an assessment of OER in the M@cMoS_2_. The OER is a four-electron water oxidation process, and the probable reaction mechanism steps are as follows [2,3]:

* + H_2_O → HO* + (H^+^ + e^−^)

HO* → O* + (H^+^ + e^−^)

O* + H_2_O → HOO* + (H^+^ + e^−^)

HOO* → * + O_2_ + (H^+^ + e^−^)

The Gibbs free energy of each step is calculated as the HER reaction in the previous part.

**Figure 7:**
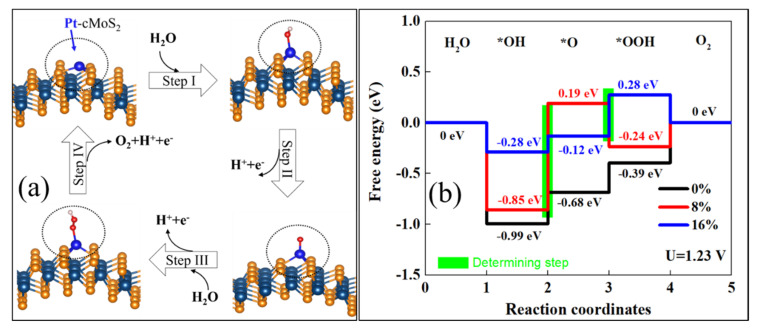
(**a**) Proposed 4e-mechanism of oxygen evolution reaction on Pt@cMoS_2_. (**b**) Gibbs free-energy diagram for the four steps of OER on Pt@cMoS_2_ at different curvatures. The green box step is the rate determining.

Figure 7a shows the optimized structure for each reaction step. The OER reaction is clearly tuned by the curvature at equilibrium hydrolysis potential (Figure 7b). We see that the enlarged curvature leads to a weak *OH adsorption of Pt@cMoS_2_. The potential determining step (PDS) is *OH oxidation (Step II) at a small curvature (≤ 8%), while PDS locates at the step *O → *OOH (Step III) at δ = 16%. The reaction rate would be 0%-Pt@cMoS_2_ > 16%-Pt@cMoS_2_ > 8%-Pt@cMoS_2_, which PDS are 0.31 eV, 0.40 eV and 1.04 eV. But the last step contains a thermal desorption of O_2_ from the slab, the production of O_2_ on 16%-Pt@cMoS_2_ would be more than that on 0%-Pt@cMoS_2_, due to the different trend between the downhill step and uphill step at Step IV for 16% and 0%, respectively. For Fe@cMoS_2_, the strong binding between *O and Fe leads to an easy oxidation of *OH (Step II, Figure S7). The determining steps are all located at Step III, and are 0.93 eV, 1.01 eV, and 1.13 eV for 0%, 8%, and 16%, respectively. The effect of curvatures on the OER performance of Fe@cMoS_2_ is similar to that of Pt@cMoS_2_. But the much larger PDS of Fe@cMoS_2_ obstacles its practical application. We also consider another pathway for *O → *OOH. For example, the *O and *OH adsorb on the metal at the same time, but the large formation energy and physical adsorption format make the pathway impossible. It means the metal decorated-cMoS_2_ has a good selectivity as an OER catalyst. Therefore, it is believed that M@cMoS_2_ would be used as a potential SAC candidate for OER if the proper anchored metals and curvatures are selected.

## 4. Conclusions

In summary, we have proposed an effective structure with an aim to activate the inert surface of TMD monolayers, which are believed to be activated by an edge effect or prior doping. We took the noble metal Pt and nonnoble metal Fe as examples. Compared with the basal MoS_2_, the curved one demonstrated a promoted ability to hold metal atoms, especially at center sites of the honeycomb structure on the crest cMoS_2_ with large curvatures. The promotion can be up to 72% for Pt@cMoS_2_. Additionally, the CINEB calculation showed the diffusion barrier increased to the point that the metal atom was stable at the H site under large compressions. Moreover, we made a series of assessments of water splitting based on the single atom anchored at cMoS_2_. The excellent HER appeared at 4%-cMoS_2_ for Pt and at 16%-cMoS_2_ for Fe, and are comparable to the traditional bulk electrode Pt/C. However, the ability of water dissociation was still inferior in our system, which indicated a limited HER performance in alkane solution. At last, the calculated OER at 16%-Pt@cMoS_2_ showed a fast reaction, due to the low PDS of 0.40 eV and the downhill step of O_2_ desorption compared with the basal plane at the equilibrium potential. Our calculations provide insightful explanations for the design of TMD-based SACs, suggesting that the curved-TMD represents a feasible approach to fabricate efficient SA water splitting electrocatalysts.

## Supplementary Materials

The following are available online at https://www.mdpi.com/article/10.3390/nano11123173/s1, Figure S1: Adsorption energy of Fe and Pt atoms at top S site on neutral cMoS_2_ as a function of compressions, Figure S2: Charge density difference of Pt@cMoS_2_ in neutral (a,d), 1e^−^ (b,e) and 2e^−^ (c,f) situations. The isosurface value is set to 0.004 e/Å^3^, Figure S3: AIMD simulation of Pt-12%cMoS_2_ as the function of simulation time. The timespan is over 4 ps, Figure S4: Diffusion coordinates of Pt atom in cMoS_2_ at different compressions, Figure S5: Optimized structures for the initial (IS, leftmost panels), transition (TS, center panels), and final (FS, rightmost panels) states of the most favorable path for the H_2_O → OH^−^ +H^+^ reaction on Fe@cMoS_2_, Figure S6: Reaction coordinate of water dissociation of Fe@cMoS_2_ at δ = 16% and the comparison with Pt bulk, Figure S7: Gibbs free-energy diagram for the four steps of OER on Fe@cMoS_2_ at different curvatures. The green box step is the rate determining, Table S1: Structure parameters of cMoS_2_ absorbed Fe atom as compressions increases, Table S2: Structure parameters of cMoS_2_ absorbed Pt atom as compressions increases, Table S3: Bader charge of Fe@cMoS_2_ at different compressions, Table S4: Bader charge of Pt@cMoS_2_ at different compressions.
