# Supplementary material for: Computational Study of the Curvature-Promoted Anchoring of Transition Metals for Water Splitting
Source: Nanomaterials (Basel). 2021 Nov 23;11(12):3173. doi: 10.3390/nano11123173 (PMC8709100; doi:10.3390/nano11123173)
Supplement: Supplementary file 1 [file nanomaterials-11-03173-s001.zip › nanomaterials-1443078-supplementary.pdf]

nanomaterials

MDPI

Supplementary Material

Computational Study of the Curvature-Promoted Anchoring of Transition Metals for Water Splitting

Weiwei Liu [1,†], Youchao Kong [1,†], Bo Wang [2], Xiaoshuang Li [2,*], Pengfei Liu [3], Alain R. Puente Santiago [4] and Tianwei He [5,*]

1 Department of Physics and Electronic Engineering, Yancheng Teachers University, Yancheng 224002, China; liuww@yctu.edu.cn (W.L.); yb87816@connect.um.edu.mo (Y.K.)
2 School of Applied Physics and Materials, Wuyi University, Jiangmen 529020, China; wangbo312@mails.ucas.ac.cn
3 Spallation Neutron Source Science Center, Institute of High Energy Physics, Chinese Academy of Sciences, Dongguan 523803, China; pfliu@ihep.ac.cn
4 Department of Chemistry and Biochemistry, University of Texas at El Paso, 500 W. University Avenue, El Paso, TX 79968, USA; arpuentesan@utep.edu
5 Fritz-Haber-Institute der Max-Planck-Gesellschaft, Faradayweg, 4-6, 14195 Berlin, Germany
* Correspondence: lixiaoshuang12@mails.ucas.ac.cn (X.L.); the@fhi-berlin.mpg.de (T.H.)
† These authors contributed equally to this work.

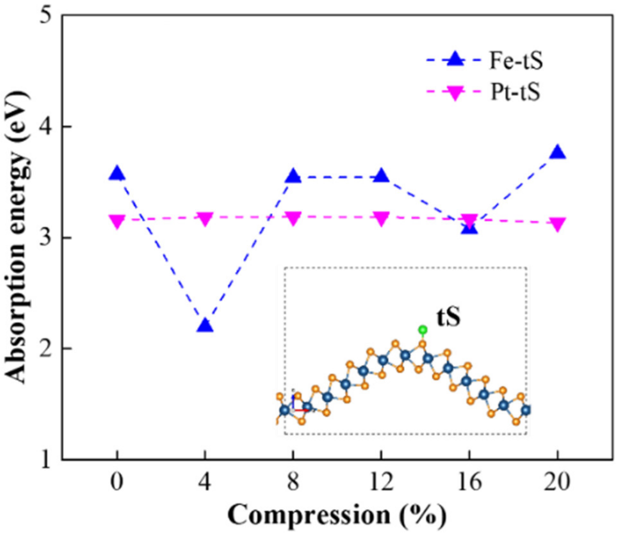

Figure S1. Adsorption energy of Fe and Pt atoms at top S site on neutral $cMoS_2$ as a function of compressions.

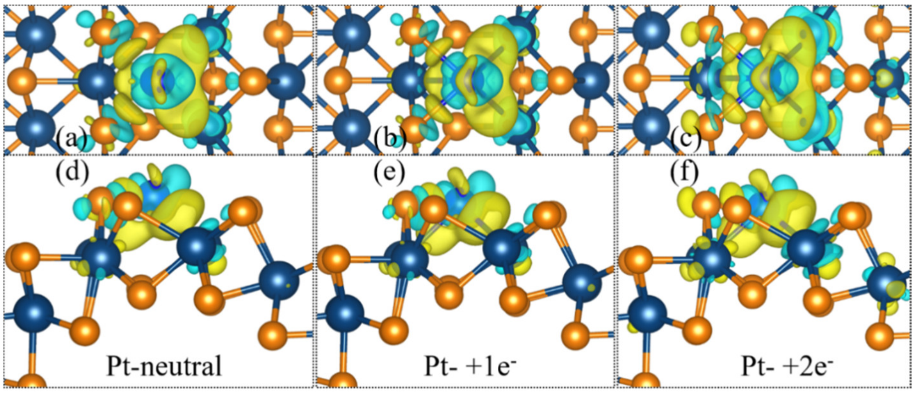

Figure S2. Charge density difference of Pt@$cMoS_2$ in neutral (**a**,**d**), $1e^-$ (**b**,**e**) and $2e^-$ (**c**,**f**) situations. The isosurface value is set to 0.004 e/Å^3.

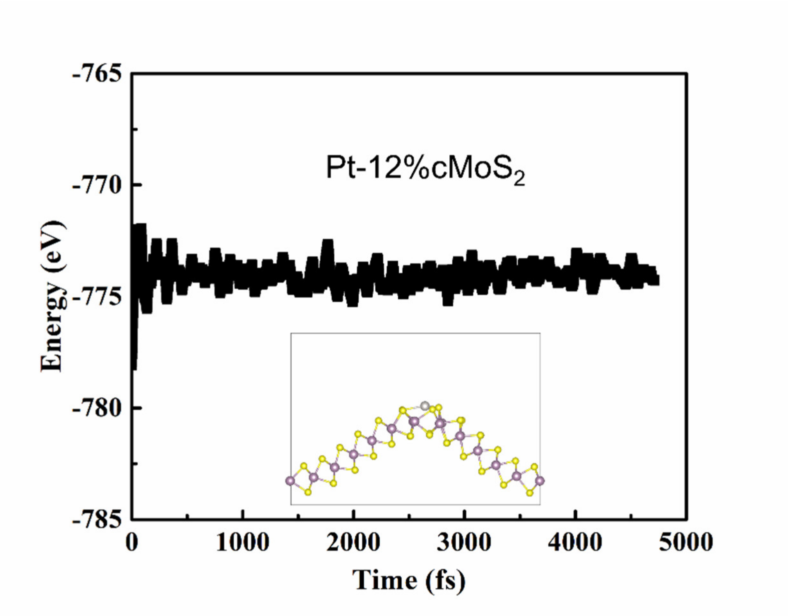

Figure S3. AIMD simulation of Pt-12%cMoS2 as the function of simulation time. The timespan is over 4 ps.

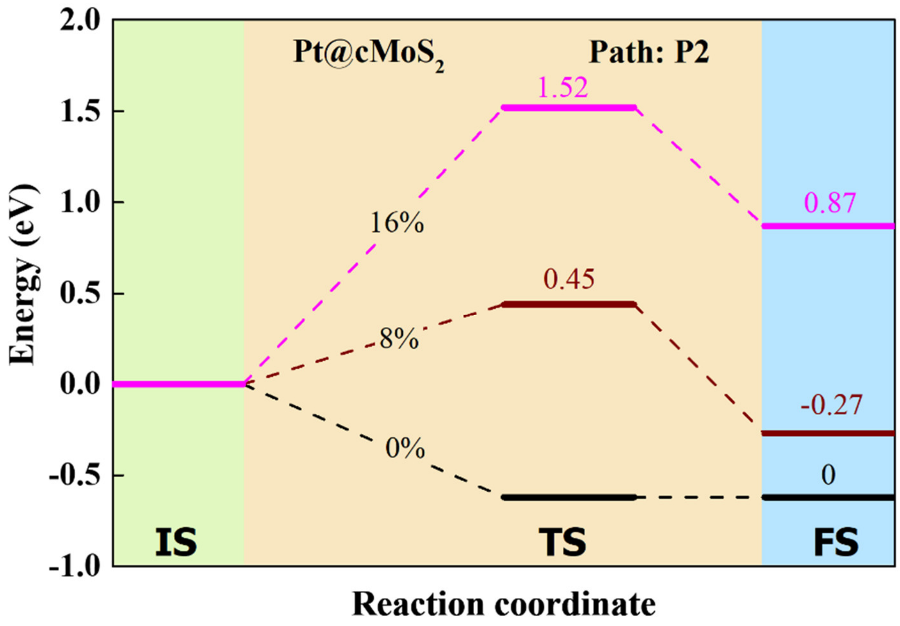

Figure S4. Diffusion coordinates of Pt atom in cMoS2 at different compressions.

Figure S5 demonstrates the optimized structure for three states of the most favorable path for $H_2O \rightarrow OH + H$ at different curvatures. It found that the H_2O molecule is favorable on the top of Fe, with the molecule approaching the Fe@cMoS2 with its O atom. The negative value of *H_2O for all cases indicates the stable absorption of H_2O on the surface of catalysts (Figure S6). The H_2O prefers to the active site of Fe on curved MoS_2 instead of Pt (100), due to the lower energy of *H_2O on cMoS2. The adsorption energy of H_2O is −0.30 eV, −0.28 eV and −0.31 eV for 0%-,8%- and 16%-Fe@cMoS2, respectively. The large curvature could enhance the adsorption ability. Figure S5 shows the optimized structure for co-adsorption of (OH + H pair) on the surfaces of cMoS2. The OH* prefer to the top of metal Fe while H* is captured by S atom near the Fe atom. Results reveal that the curvature has little effect on the adsorption of OH-H pair. In comparison, Pt (100) surface shows a rela-

tively low adsorption energy, which means the large possibility for holding water dissociation product (H^+). Then we performed CINEB to evaluate the transition state (TS) from initial state (IS) to final state (FS). The activation energy barrier ($E_{barrier}$) is calculated as $E_{barrier}$ = E(TS) − E(IS). The calculated $E_{barrier}$ on the surface of Fe@cMoS$_2$ is 1.38eV, 2.4 eV,1.55eV for 0%,8% and 16%, respectively. The $E_{barrier}$ is much larger than Pt (100) of 0.78eV and MoS2-mo-edge [34], which means the curved cMoS$_2$ is sluggish for water splitting in alkane environment.

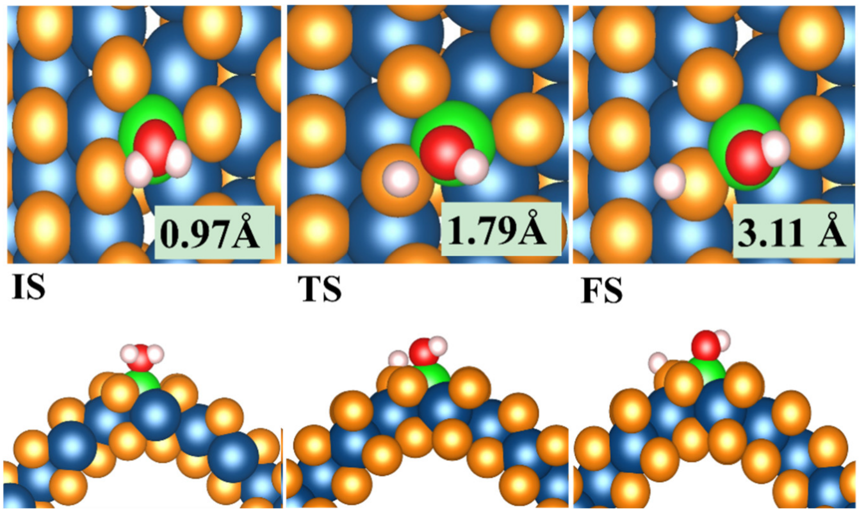

Figure S5. Optimized structures for the initial (IS, leftmost panels), transition (TS, center panels), and final (FS, rightmost panels) states of the most favorable path for the $H_2O \rightarrow OH^- + H^+$ reaction on Fe@cMoS$_2$.

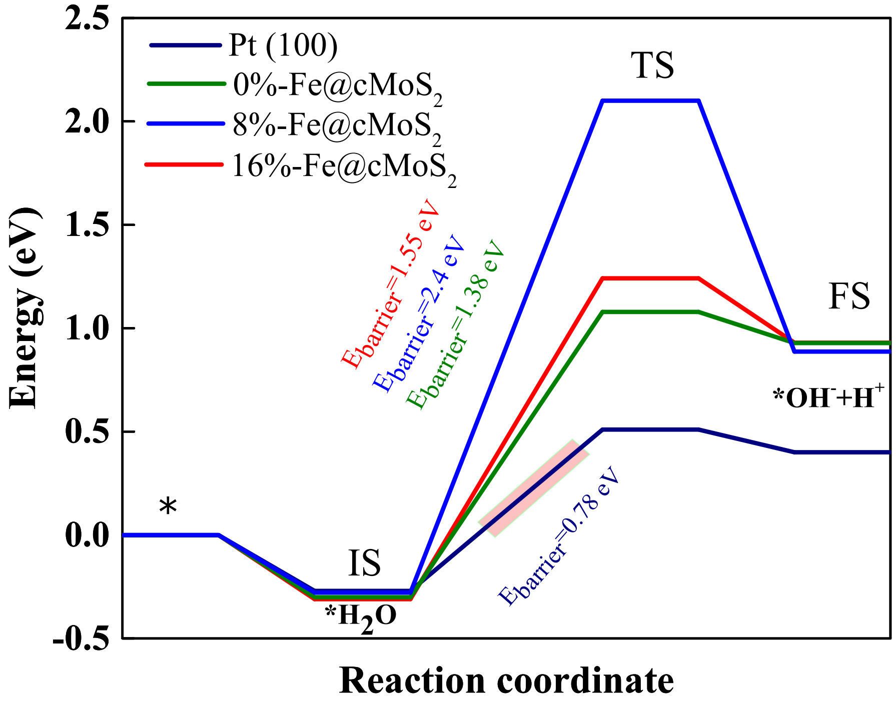

Figure S6. Reaction coordinate of water dissociation of Fe@cMoS$_2$ at δ = 16% and the comparison with Pt bulk.

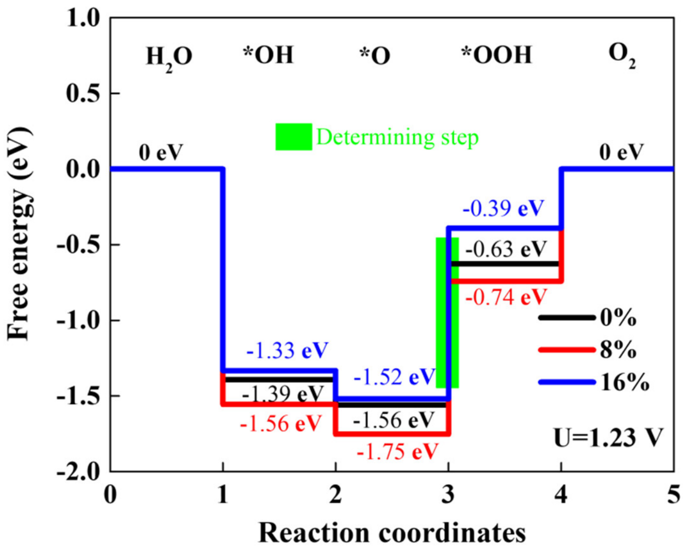

Figure S7. Gibbs free-energy diagram for the four steps of OER on Fe@cMoS_2 at different curvatures. The green box step is the rate determining.

Table S1. Structure parameters of cMoS_2 absorbed Fe atom as compressions increases.

Compression (%)	Distance of S-Fe (Å)			Distance of Fe-Mo (Å)	Angle (°)
δ	a1	a2	a3	b1	θS-Mo-S
0	2.10	2.10	2.10	2.94	83.57
4	2.12	2.10	2.10	2.93	84.69
8	2.14	2.16	2.16	2.66	92.17
12	2.31	2.17	2.17	2.48	97.84
16	2.44	2.22	2.21	2.4	103.23
20	2.59	2.27	2.27	2.4	107.55

Table S2. Structure parameters of cMoS_2 absorbed Pt atom as compressions increases.

Compression (%)	Distance of S-Pt (Å)			Distance of Pt-Mo (Å)	Angle (°)
δ	a1	a2	a3	b1	θS-Mo-S
0	2.35	2.35	2.35	3.54	82.82
4	2.34	2.32	2.32	3.45	83.91
8	2.29	2.28	2.28	2.9	91.84
12	2.70	2.31	2.31	2.73	100.39
16	3.08	2.35	2.35	2.73	107.03
20	3.28	2.38	2.38	2.76	112.30

Table S3. Bader charge of Fe@cMoS$_2$ at different compressions.

Bader Transfer (e): Fe@cMoS$_2$			
**Compression (%)**	**Neutral**	**+1e$^-$**	**+2e$^-$**
0	7.362	7.465	7.529
4	7.362	7.482	7.547
8	7.468	7.502	7.588
12	7.616	7.602	7.631
16	7.757	7.672	7.684
20	7.645	7.654	7.682

Table S4. Bader charge of Pt@cMoS$_2$ at different compressions.

Pt Bader Transfer (e): Pt@cMoS$_2$			
**Compression (%)**	**Neutral**	**+1e$^-$**	**+2e$^-$**
0	9.913	10.063	10.153
4	9.908	10.074	10.182
8	9.999	10.074	10.133
12	10.228	10.267	10.313
16	10.254	10.317	10.339
20	10.284	--	--

The structure of Planar MoS_2

```
   1.00000000000000
    33.0942993163999972    0.0000000000000000    0.0000000000000000
     0.0000000000000000    9.5535001755000000    0.0000000000000000
     0.0000000000000000    0.0000000000000000   20.0000000000000000
Direct
  0.1643768449764571 -0.0000000000000000  0.4135927848483881
  0.2477121114270434  0.1666680767459661  0.4135863448514414
  0.3310445460864407 -0.0000000000000000  0.4135871877262204
  0.4143772692000879  0.1666675324538868  0.4135874782315208
  0.4977093998216757 -0.0000000000000000  0.4135936225721746
  0.5810444333368083  0.1666660035670754  0.4135911533508846
  0.6643768445353522 -0.0000000000000000  0.4135927875326765
  0.7477121171129130  0.1666680789111462  0.4135863432597688
  0.8310445559359059 -0.0000000000000000  0.4135871800679127
  0.9143772719743382  0.1666675301417523  0.4135874751127214
  0.9977093774952505 -0.0000000000000000  0.4135936269458281
  0.0810444235046875  0.1666660072076619  0.4135911532184040
  0.1643783464576880  0.3333327852505537  0.4135899088540362
  0.2477105173309441  0.5000000000000000  0.4135855313881363
  0.3310453758896598  0.3333332476072356  0.4135882936695454
  0.4143769081782683  0.5000000000000000  0.4135908419166422
  0.4977108885784924  0.3333345920266143  0.4135917795347179
  0.5810439302413786  0.5000000000000000  0.4135922836363423
  0.6643783466513039  0.3333327866837468  0.4135899090182568
  0.7477105230988529  0.5000000000000000  0.4135855292690705
  0.8310453847783646  0.3333332493510852  0.4135882864511417
  0.9143769114694597  0.5000000000000000  0.4135908398566723
  0.9977108687196614  0.3333345951232946  0.4135917858458935
  0.0810439185781820  0.5000000000000000  0.4135922860949622
  0.1643783464576880  0.6666672437494451  0.4135899088540362
  0.2477121114270434  0.8333318942540350  0.4135863448514414
  0.3310453758896598  0.6666667813927633  0.4135882936695454
  0.4143772692000879  0.8333324385461144  0.4135874782315208
  0.4977108885784924  0.6666654369733845  0.4135917795347179
  0.5810444333368083  0.8333339674329258  0.4135911533508846
  0.6643783466513039  0.6666672423162520  0.4135899090182568
  0.7477121171129130  0.8333318920888549  0.4135863432597688
  0.8310453847783646  0.6666667796489137  0.4135882864511417
  0.9143772719743382  0.8333324408582489  0.4135874751127214
  0.9977108687196614  0.6666654338767043  0.4135917858458935
  0.0810444235046875  0.8333339637923393  0.4135911532184040
  0.2199299016686148 -0.0000000000000000  0.4916598629429184
  0.1365895667550482  0.1666811104639301  0.4916689218253270
  0.2199283181390901 -0.0000000000000000  0.3355201921009775
  0.1365936585739067  0.1666878317490584  0.3355249757598711
  0.3865887223428511 -0.0000000000000000  0.4916507984203457
  0.3032652122664076  0.1666756787958196  0.4916634597262305
  0.3865993817539542 -0.0000000000000000  0.3355200051461499
```

0.3032627898846871	0.1666873347835558	0.3355243541144650
0.5532651705486965	-0.0000000000000000	0.4916504520712947
0.4699245953551556	0.1666845736711841	0.4916571684335766
0.5532573435109174	-0.0000000000000000	0.3355266935842685
0.4699291223862819	0.1666679316743361	0.3355291704413398
0.7199299025625060	-0.0000000000000000	0.4916598677687328
0.6365895629227004	0.1666811118682752	0.4916689054463719
0.7199283197019360	-0.0000000000000000	0.3355201878858390
0.6365936544853088	0.1666878365866323	0.3355249919642814
0.8865887158601647	-0.0000000000000000	0.4916507913902217
0.8032652207169279	0.1666756773018680	0.4916634628263640
0.8865993931178205	-0.0000000000000000	0.3355200102887882
0.8032627995496101	0.1666873330762424	0.3355243498715940
0.0532651485739048	-0.0000000000000000	0.4916504466045991
0.9699245782779857	0.1666845709406323	0.4916571710456027
0.0532573224556958	-0.0000000000000000	0.3355266992146801
0.9699291222440810	0.1666679290670635	0.3355291685755754
0.2199297989064876	0.3333132622996198	0.4916588718107678
0.1365947431268213	0.5000000000000000	0.4916574127842408
0.2199283180339624	0.3333183623315858	0.3355024780719710
0.1365951672473709	0.5000000000000000	0.3355282496464753
0.3865915348614963	0.3333108013377968	0.4916533951442345
0.3032618898882224	0.5000000000000000	0.4916487557035208
0.3865944483316275	0.3333276838410610	0.3355226527975056
0.3032590282766445	0.5000000000000000	0.3355280403542630
0.5532614426625175	0.3333104523489314	0.4916524587328394
0.4699242090063027	0.5000000000000000	0.4916649393336163
0.5532638766987874	0.3333233787892794	0.3355271752322571
0.4699285513234121	0.5000000000000000	0.3355224907238614
0.7199297992296140	0.3333132640935768	0.4916588746205238
0.6365947392621724	0.5000000000000000	0.4916573969169831
0.7199283186395864	0.3333183654922576	0.3355024746365680
0.6365951631976255	0.5000000000000000	0.3355282665391456
0.8865915286510899	0.3333107992617757	0.4916533914893093
0.8032618971812543	0.5000000000000000	0.4916487584002650
0.8865944602667987	0.3333276832563198	0.3355226554717256
0.8032590368298027	0.5000000000000000	0.3355280354686706
0.0532614208660862	0.3333104549561614	0.4916524478198053
0.9699241926062516	0.5000000000000000	0.4916649382371636
0.0532638553724582	0.3333233798221527	0.3355271858453071
0.9699285519304571	0.5000000000000000	0.3355224924143847
0.2199297989064876	0.6666867667003791	0.4916588718107678
0.1365895667550482	0.8333188605360711	0.4916689218253270
0.2199283180339624	0.6666816666684131	0.3355024780719710
0.1365936585739067	0.8333121392509427	0.3355249757598711
0.3865915348614963	0.6666892276622021	0.4916533951442345
0.3032652122664076	0.8333242922041816	0.4916634597262305
0.3865944483316275	0.6666723451589378	0.3355226527975056
0.3032627898846871	0.8333126362164454	0.3355243541144650

0.5532614426625175	0.6666895766510674	0.4916524587328394
0.4699245953551556	0.8333153973288171	0.4916571684335766
0.5532638766987874	0.6666766502107195	0.3355271752322571
0.4699291223862819	0.8333320393256650	0.3355291704413398
0.7199297992296140	0.6666867649064221	0.4916588746205238
0.6365895629227004	0.8333188591317260	0.4916689054463719
0.7199283186395864	0.6666816635077414	0.3355024746365680
0.6365936544853088	0.8333121344133688	0.3355249919642814
0.8865915286510899	0.6666892297382232	0.4916533914893093
0.8032652207169279	0.8333242936981332	0.4916634628263640
0.8865944602667987	0.6666723457436790	0.3355226554717256
0.8032627995496101	0.8333126379237588	0.3355243498715940
0.0532614208660862	0.6666895740438374	0.4916524478198053
0.9699245782779857	0.8333154000593689	0.4916571710456027
0.0532638553724582	0.6666766491778462	0.3355271858453071
0.9699291222440810	0.8333320419329376	0.3355291685755754

The structure of 4%-MoS_2

1.00000000000000

31.7705001831000011	0.0000000000000000	0.0000000000000000
0.0000000000000000	9.5535001755000000	0.0000000000000000
0.0000000000000000	0.0000000000000000	20.0000000000000000

Direct

0.9996444120548399	-0.0000000000000000	0.2670686885159266
0.0835643843271965	0.1666573448821627	0.2716738978516677
0.1669148186438259	-0.0000000000000000	0.2866511581026588
0.2497586485533598	0.1666597055727673	0.3069449250317800
0.3325806604995477	-0.0000000000000000	0.3272751412419084
0.4158775092548858	0.1666546544423757	0.3429066276083755
0.4997154979823478	-0.0000000000000000	0.3496054168263659
0.5836034946442501	0.1666748498917610	0.3453259954289849
0.6670022610841067	-0.0000000000000000	0.3315359116345673
0.7498945884736439	0.1666592359127598	0.3120937016472459
0.8326501267384702	-0.0000000000000000	0.2914447507602974
0.9158240069733968	0.1666633771956620	0.2746798389488610
0.9996389192136992	0.3333287698514151	0.2670717858219460
0.0835659813482037	0.5000000000000000	0.2716778543713903
0.1669186822019703	0.3333314007328896	0.2866595898645184
0.2497511263333816	0.5000000000000000	0.3069524523940017
0.3325845622377508	0.3333349202515490	0.3272836442254074
0.4158706523418829	0.5000000000000000	0.3429066581744277
0.4997178228642961	0.3333223413358321	0.3496127676570350
0.5836023031672319	0.5000000000000000	0.3453192711522368
0.6670108368665103	0.3333412913857878	0.3315412729561368
0.7498961679784687	0.5000000000000000	0.3121028999844898
0.8326518315779540	0.3333510917327845	0.2914457767339905
0.9158247108621689	0.5000000000000000	0.2746703578389707
0.9996389192136992	0.6666712591485766	0.2670717858219460
0.0835643843271965	0.8333426261178385	0.2716738978516677
0.1669186822019703	0.6666686282671093	0.2866595898645184
0.2497586485533598	0.8333402654272337	0.3069449250317800
0.3325845622377508	0.6666651087484499	0.3272836442254074
0.4158775092548858	0.8333453165576254	0.3429066276083755
0.4997178228642961	0.6666776876641667	0.3496127676570350
0.5836034946442501	0.8333251211082401	0.3453259954289849
0.6670108368665103	0.6666587376142111	0.3315412729561368
0.7498945884736439	0.8333407350872414	0.3120937016472459
0.8326518315779540	0.6666489372672144	0.2914457767339905
0.9158240069733968	0.8333365938043391	0.2746798389488610
0.0541494382734538	-0.0000000000000000	0.3481364197771670
0.9745034935059287	0.1666663709559313	0.3475052745880053
0.0578019956848214	-0.0000000000000000	0.1903707873944967
0.9695377871089975	0.1666691345008016	0.1898573143937523
0.2152235342842563	-0.0000000000000000	0.3783024722225525
0.1340875533326765	0.1666684904800329	0.3597012734108689
0.2298286851409701	-0.0000000000000000	0.2219674178349000

0.1449950017349148	0.1666982960733962	0.2026638558084298
0.3829773198157509	-0.0000000000000000	0.4168193619870331
0.2980836067870535	0.1666855211548204	0.3989878606510813
0.3938767400720865	-0.0000000000000000	0.2598153671473553
0.3125603235122907	0.1666978527679587	0.2426227958672417
0.5577438095247307	-0.0000000000000000	0.4264213666760762
0.4699107551363122	0.1666757779532389	0.4270669903767600
0.5543165655424664	-0.0000000000000000	0.2686245990946780
0.4742988185427256	0.1666885794710657	0.2693512025608613
0.7296878485749575	-0.0000000000000000	0.3969365430034102
0.6446730319188803	0.1666878562821164	0.4151940977605191
0.7156258585886051	-0.0000000000000000	0.2404575179496377
0.6345577601337378	0.1666955368225293	0.2580188475670623
0.8942491645893419	-0.0000000000000000	0.3581078836789104
0.8127830252478582	0.1666956003700087	0.3762742834680731
0.8825885046527251	-0.0000000000000000	0.2012082001624210
0.7980672746296038	0.1666981381439457	0.2199371430300513
0.0541465909856969	0.3333283334219589	0.3481239073728893
0.9744993904339634	0.5000000000000000	0.3474983556645747
0.0577973216259128	0.3333119218321685	0.1904053470912394
0.9695478732945544	0.5000000000000000	0.1898528810934254
0.2152273912183243	0.3333000849278276	0.3783117434390387
0.1340937798823995	0.5000000000000000	0.3597077830347484
0.2298242140377868	0.3333093378871563	0.2219707995760736
0.1450029354684075	0.5000000000000000	0.2026793274143721
0.3829767957152760	0.3332845420313004	0.4168248313195361
0.2980741758952509	0.5000000000000000	0.3990079818004672
0.3938805100259083	0.3333033613188899	0.2598166154870598
0.3125556331782021	0.5000000000000000	0.2426556383080730
0.5577362724745999	0.3333339509865136	0.4264281999914635
0.4699019429477400	0.5000000000000000	0.4270569935333769
0.5543143190696079	0.3333099903908456	0.2686073219708928
0.4743032540733175	0.5000000000000000	0.2693786861571785
0.7297001032977325	0.3332909755725731	0.3969493938241715
0.6446720896087595	0.5000000000000000	0.4152048336016347
0.7156368364993565	0.3333166327324719	0.2404612600192498
0.6345602622988432	0.5000000000000000	0.2580072767514868
0.8942450779071033	0.3333213714597306	0.3580967449930275
0.8127853358787660	0.5000000000000000	0.3763102798567841
0.8825955988134453	0.3333185452633275	0.2011886448790975
0.7980556033598892	0.5000000000000000	0.2199403574131902
0.0541465909856969	0.6666716955780400	0.3481239073728893
0.9745034935059287	0.8333336000440699	0.3475052745880053
0.0577973216259128	0.6666881071678303	0.1904053470912394
0.9695377871089975	0.8333308364991996	0.1898573143937523
0.2152273912183243	0.6666999440721713	0.3783117434390387
0.1340875533326765	0.8333314805199682	0.3597012734108689
0.2298242140377868	0.6666906911128426	0.2219707995760736
0.1449950017349148	0.8333016749266050	0.2026638558084298

0.3829767957152760	0.6667154869686984	0.4168248313195361
0.2980836067870535	0.8333144498451809	0.3989878606510813
0.3938805100259083	0.6666966676811090	0.2598166154870598
0.3125603235122907	0.8333021182320425	0.2426227958672417
0.5577362724745999	0.6666660780134852	0.4264281999914635
0.4699107551363122	0.8333241930467623	0.4270669903767600
0.5543143190696079	0.6666900386091532	0.2686073219708928
0.4742988185427256	0.8333113915289354	0.2693512025608613
0.7297001032977325	0.6667090534274256	0.3969493938241715
0.6446730319188803	0.8333121147178848	0.4151940977605191
0.7156368364993565	0.6666833962675269	0.2404612600192498
0.6345577601337378	0.8333044341774719	0.2580188475670623
0.8942450779071033	0.6666786575402682	0.3580967449930275
0.8127830252478582	0.8333043706299923	0.3762742834680731
0.8825955988134453	0.6666814837366714	0.2011886448790975
0.7980672746296038	0.8333018328560555	0.2199371430300513

The structure of 8%-MoS_2
1.00000000000000

30.4468002318999993	0.0000000000000000	0.0000000000000000
0.0000000000000000	9.5535001755000000	0.0000000000000000
0.0000000000000000	0.0000000000000000	20.0000000000000000

Direct

0.9991730861272795	-0.0000000000000000	0.1882457723681629
0.0877461553712682	0.1666761887866338	0.2014260269335834
0.1716537896054652	-0.0000000000000000	0.2435454297694085
0.2504005197130916	0.1666520572009874	0.3022394851893213
0.3285396040436638	-0.0000000000000000	0.3626285365908589
0.4112464646177581	0.1666610054792815	0.4095480776652150
0.4992018682945744	-0.0000000000000000	0.4296488540120242
0.5877493461464049	0.1666643573782066	0.4162999612030382
0.6716162251080771	-0.0000000000000000	0.3741596590310979
0.7503686916191911	0.1666684146011468	0.3155924832687123
0.8285392433193812	-0.0000000000000000	0.2553714513298281
0.9112365213373715	0.1666608528794158	0.2084747654930380
0.9991750036209888	0.3333315473839477	0.1882558084533941
0.0877341368930736	0.5000000000000000	0.2014351057112314
0.1716521616464896	0.3333433882372827	0.2435485162950520
0.2503908347877563	0.5000000000000000	0.3022464398664370
0.3285405886331404	0.3333358752343866	0.3626419032306621
0.4112423696050824	0.5000000000000000	0.4095469853995304
0.4992048930076081	0.3333359288657928	0.4296655020122546
0.5877434862366187	0.5000000000000000	0.4163160601492202
0.6716104683064122	0.3333318104141831	0.3741535378567440
0.7503635721510874	0.5000000000000000	0.3155890879913210
0.8285383349062637	0.3333323891551001	0.2553613503968031
0.9112317917804243	0.5000000000000000	0.2084775962045848
0.9991750036209888	0.6666684816160511	0.1882558084533941
0.0877461553712682	0.8333237822133744	0.2014260269335834
0.1716521616464896	0.6666566407627161	0.2435485162950520
0.2504005197130916	0.8333479137990137	0.3022394851893213
0.3285405886331404	0.6666641537656121	0.3626419032306621
0.4112464646177581	0.8333389655207196	0.4095480776652150
0.4992048930076081	0.6666641001342062	0.4296655020122546
0.5877493461464049	0.8333356136217945	0.4162999612030382
0.6716104683064122	0.6666682185858158	0.3741535378567440
0.7503686916191911	0.8333315563988544	0.3155924832687123
0.8285383349062637	0.6666676398448989	0.2553613503968031
0.9112365213373715	0.8333391181205854	0.2084747654930380
0.0532580412054735	-0.0000000000000000	0.2723904114241886
0.9766194031736323	0.1666770148767113	0.2699720460589069
0.0641368893688651	-0.0000000000000000	0.1168767939716171
0.9631520828095004	0.1667050711302638	0.1149604407323224
0.2026755446702664	-0.0000000000000000	0.3539091109287805
0.1284693493683611	0.1666913121851755	0.3027742963484625
0.2468033408014642	-0.0000000000000000	0.2108367004620498

0.1604153662096016	0.1666855636929513	0.1532269311702228
0.3666451101644700	-0.0000000000000000	0.4700137282496446
0.2802320482817540	0.1666865527917538	0.4146067186902394
0.4004239222505700	-0.0000000000000000	0.3215168591231787
0.3250516469055963	0.1666913963869285	0.2721180123063802
0.5642161395267038	-0.0000000000000000	0.5008906580812356
0.4632205093377424	0.1666789044759186	0.5029855204632604
0.5532156537078838	-0.0000000000000000	0.3453829992851696
0.4765993354878753	0.1667000096894870	0.3479721103007943
0.7467446504332987	-0.0000000000000000	0.4069809615493980
0.6603937026168941	0.1666734032472039	0.4644806748936594
0.7026805693944439	-0.0000000000000000	0.2638425983571242
0.6284348586751707	0.1666856185333190	0.3148948987127155
0.9004226378471591	-0.0000000000000000	0.2965196172601358
0.8249931726426016	0.1666878559303662	0.3458880901885222
0.8666561194439573	-0.0000000000000000	0.1480159740614848
0.7802715640486965	0.1666786338300350	0.2032916097487735
0.0532552704746089	0.3333247291048520	0.2724153906816718
0.9766192813432664	0.5000000000000000	0.2699755473505008
0.0641215916706968	0.3332883735928820	0.1168830479678882
0.9631455799715767	0.5000000000000000	0.1149860994340440
0.2026770516696695	0.3332819875329322	0.3539164276178711
0.1284676992420885	0.5000000000000000	0.3027964861701430
0.2468000929330006	0.3333033359446392	0.2108428097481298
0.1603841448031546	0.5000000000000000	0.1532315730638576
0.3666641451682738	0.3332995439407501	0.4700219676283925
0.2802149447808535	0.5000000000000000	0.4146166690827254
0.4004263385828396	0.3333009767218300	0.3215212406744329
0.3250334128833717	0.5000000000000000	0.2721346908510981
0.5642213095738112	0.3333028701515482	0.5009111055077967
0.4632221845581495	0.5000000000000000	0.5029976414046827
0.5532136129999102	0.3333325883640441	0.3454052049311993
0.4765907509180892	0.5000000000000000	0.3479914047051843
0.7467203013811569	0.3333333391335166	0.4069800180484160
0.6603916241251404	0.5000000000000000	0.4644609740363868
0.7026721207983062	0.3333057255424229	0.2638317095464095
0.6284235233101212	0.5000000000000000	0.3149182587247631
0.9004161255254209	0.3333108247153775	0.2965010949653644
0.8249802631403761	0.5000000000000000	0.3458952836331055
0.8666561805821659	0.3333090394689546	0.1479953146400046
0.7802752894702515	0.5000000000000000	0.2032900962264915
0.0532552704746089	0.6666752998951470	0.2724153906816718
0.9766194031736323	0.8333229561232900	0.2699720460589069
0.0641215916706968	0.6667116554071170	0.1168830479678882
0.9631520828095004	0.8332948998697374	0.1149604407323224
0.2026770516696695	0.6667180414670668	0.3539164276178711
0.1284693493683611	0.8333086588148328	0.3027742963484625
0.2468000929330006	0.6666966930553596	0.2108428097481298
0.1604153662096016	0.8333144073070499	0.1532269311702228

0.3666641451682738	0.6667004850592488	0.4700219676283925
0.2802320482817540	0.8333134182082473	0.4146067186902394
0.4004263385828396	0.6666990522781689	0.3215212406744329
0.3250516469055963	0.8333085746130727	0.2721180123063802
0.5642213095738112	0.6666971588484507	0.5009111055077967
0.4632205093377424	0.8333210665240827	0.5029855204632604
0.5532136129999102	0.6666674406359546	0.3454052049311993
0.4765993354878753	0.8332999613105142	0.3479721103007943
0.7467203013811569	0.6666666898664821	0.4069800180484160
0.6603937026168941	0.8333265677527972	0.4644806748936594
0.7026721207983062	0.6666943034575760	0.2638317095464095
0.6284348586751707	0.8333143524666821	0.3148948987127155
0.9004161255254209	0.6666892042846213	0.2965010949653644
0.8249931726426016	0.8333121150696350	0.3458880901885222
0.8666561805821659	0.6666909895310442	0.1479953146400046
0.7802715640486965	0.8333213371699660	0.2032916097487735

The structure of 12-MoS_2
1.00000000000000

29.1229991912999999	0.0000000000000000	0.0000000000000000
0.0000000000000000	9.5535001755000000	0.0000000000000000
0.0000000000000000	0.0000000000000000	20.0000000000000000

Direct

0.9985479890912217	0.0000000000000000	0.1439283869661560
0.0922536806487148	0.1666634440375270	0.1631024571105551
0.1762955242396935	0.0000000000000000	0.2216823070983560
0.2510255502304672	0.1666596133939324	0.2996144319419188
0.3246233627968357	0.0000000000000000	0.3795238441978260
0.4062315423171063	0.1666708394632452	0.4443763350299875
0.4985898506464956	0.0000000000000000	0.4739434825478326
0.5923344973705558	0.1666543061399997	0.4549048905558216
0.6763913317806459	0.0000000000000000	0.3962924597705899
0.7510471996458282	0.1666719572962996	0.3182368799554571
0.8245910446670516	0.0000000000000000	0.2382391569256665
0.9061803392906398	0.1666628903680915	0.1733741141670412
0.9985513523360581	0.3333324486820525	0.1439295124960316
0.0922511527090170	0.5000000000000000	0.1631055168453579
0.1763003844898128	0.3333327518608724	0.2216773048595500
0.2510213302652625	0.5000000000000000	0.2996072448009049
0.3246242884175660	0.3333311136190527	0.3795249095885969
0.4062266614511140	0.5000000000000000	0.4443723383272814
0.4985946819752770	0.3333385969193313	0.4739386703861509
0.5923393159684578	0.5000000000000000	0.4549024186212825
0.6763953954704141	0.3333273590680375	0.3962941014842934
0.7510421589966589	0.5000000000000000	0.3182381451473409
0.8245904329062668	0.3333340347794494	0.2382297151125677
0.9061835266889831	0.5000000000000000	0.1733686707888554
0.9985513523360581	0.6666675803179463	0.1439295124960316
0.0922536806487148	0.8333365269624742	0.1631024571105551
0.1763003844898128	0.6666672771391265	0.2216773048595500
0.2510255502304672	0.8333403576060687	0.2996144319419188
0.3246242884175660	0.6666689153809461	0.3795249095885969
0.4062315423171063	0.8333291315367558	0.4443763350299875
0.4985946819752770	0.6666614320806676	0.4739386703861509
0.5923344973705558	0.8333456648600015	0.4549048905558216
0.6763953954704141	0.6666726699319614	0.3962941014842934
0.7510471996458282	0.8333280137037016	0.3182368799554571
0.8245904329062668	0.6666659942205494	0.2382297151125677
0.9061803392906398	0.8333370806319097	0.1733741141670412
0.0535470089277400	0.0000000000000000	0.2297541012176259
0.9772498408392818	0.1666776079601280	0.2260516035402614
0.0697343321277631	0.0000000000000000	0.0768030794250762
0.9575430685932672	0.1666752670864360	0.0741638270383362
0.1961613116926274	0.0000000000000000	0.3386256708065458
0.1264477011541441	0.1667003548985861	0.2712378439853911
0.2574228319046255	0.0000000000000000	0.2082821577008288

0.1722592154154678	0.1666755631053011	0.1295728303111953
0.3542810921983408	0.0000000000000000	0.4948873597875120
0.2689704368508019	0.1666840716395153	0.4185898645144110
0.4025910601303042	0.0000000000000000	0.3552880588847152
0.3311247256571412	0.1666642388272108	0.2892708449845237
0.5697425981386151	0.0000000000000000	0.5411512969326053
0.4575356125355537	0.1666877841240012	0.5436384992234955
0.5536469694642484	0.0000000000000000	0.3882066801853271
0.4773385456163394	0.1666850914746509	0.3917993224758824
0.7575381812437219	0.0000000000000000	0.4095284640023721
0.6723723811863546	0.1666836220334931	0.4883995290527171
0.6961553555563841	0.0000000000000000	0.2793288086508258
0.6265285391674318	0.1666654896784365	0.3467754602513915
0.9025510716037273	0.0000000000000000	0.2624707564692899
0.8311448218390519	0.1666956214948578	0.3284649486199682
0.8542313119656336	0.0000000000000000	0.1228410839356876
0.7689125389756686	0.1666773212931613	0.1992553157315541
0.0535488678782111	0.3333206355877179	0.2297587910975371
0.9772538572482199	0.5000000000000000	0.2260554957826012
0.0697460438878080	0.3333076066068375	0.0768250667371394
0.9575389839193986	0.5000000000000000	0.0741559593745241
0.1961558020574886	0.3333118707509571	0.3386108534517108
0.1264518236392080	0.5000000000000000	0.2712422548584688
0.2574338777105869	0.3332969735615940	0.2082928187130386
0.1722608861242396	0.5000000000000000	0.1295792238679912
0.3542805609856700	0.3333032727219828	0.4949045736024883
0.2689578801021180	0.5000000000000000	0.4185702590665083
0.4025868952869421	0.3333175531698190	0.3552723066495824
0.3311178164037550	0.5000000000000000	0.2892765121097836
0.5697552614929688	0.3333223048544908	0.5411429719361882
0.4575490580626044	0.5000000000000000	0.5436230080999053
0.5536579596679692	0.3333071032903569	0.3882177091749720
0.4773267304931681	0.5000000000000000	0.3917841411733783
0.7575425278493804	0.3333139601566396	0.4095394697050579
0.6723840794159278	0.5000000000000000	0.4884105937439218
0.6961466873065282	0.3333052869924899	0.2793207421804944
0.6265436376678459	0.5000000000000000	0.3467814094805761
0.9025448910113079	0.3333348910265188	0.2624496312977943
0.8311417342322452	0.5000000000000000	0.3284500317153130
0.8542395214400332	0.3333251294007556	0.1228295758192328
0.7689157304279134	0.5000000000000000	0.1992639921264598
0.0535488678782111	0.6666793934122811	0.2297587910975371
0.9772498408392818	0.8333223630398733	0.2260516035402614
0.0697460438878080	0.6666924223931613	0.0768250667371394
0.9575430685932672	0.8333247039135652	0.0741638270383362
0.1961558020574886	0.6666881582490418	0.3386108534517108
0.1264477011541441	0.8332996161014151	0.2712378439853911
0.2574338777105869	0.6667030554384048	0.2082928187130386
0.1722592154154678	0.8333244078947001	0.1295728303111953

0.3542805609856700	0.6666967562780161	0.4949045736024883
0.2689704368508019	0.8333158993604858	0.4185898645144110
0.4025868952869421	0.6666824758301798	0.3552723066495824
0.3311247256571412	0.8333357321727902	0.2892708449845237
0.5697552614929688	0.6666777241455081	0.5411429719361882
0.4575356125355537	0.8333121868759998	0.5436384992234955
0.5536579596679692	0.6666929257096420	0.3882177091749720
0.4773385456163394	0.8333148795253502	0.3917993224758824
0.7575425278493804	0.6666860688433592	0.4095394697050579
0.6723723811863546	0.8333163489665081	0.4883995290527171
0.6961466873065282	0.6666947420075090	0.2793207421804944
0.6265285391674318	0.8333344813215647	0.3467754602513915
0.9025448910113079	0.6666651379734801	0.2624496312977943
0.8311448218390519	0.8333043495051433	0.3284649486199682
0.8542395214400332	0.6666748995992433	0.1228295758192328
0.7689125389756686	0.8333226497068398	0.1992553157315541

The structure of 16-MoS_2
1.00000000000000

27.7992000580000003	0.0000000000000000	0.0000000000000000
0.0000000000000000	9.5535001755000000	0.0000000000000000
0.0000000000000000	0.0000000000000000	20.0000000000000000

Direct

0.9978581258227072	-0.0000000000000000	0.1067543236056559
0.0979584996892967	0.1666543748443453	0.1312798111277074
0.1821254961914406	-0.0000000000000000	0.2039977385065428
0.2517834751752839	0.1666633988673851	0.2977911339937778
0.3197167591002302	-0.0000000000000000	0.3936221386373976
0.3999967118425269	0.1666685119961812	0.4734861465755246
0.4978576254528840	-0.0000000000000000	0.5111358688194589
0.5979761406057371	0.1666786559904792	0.4865845014338975
0.6821343713495828	-0.0000000000000000	0.4138575409342821
0.7517651600991582	0.1666654613292447	0.3200390197325302
0.8197213507240613	-0.0000000000000000	0.2242428712577922
0.9000077069439324	0.1666685114827420	0.1444116860188132
0.9978606521275569	0.3333269034513245	0.1067581080156540
0.0979522071173942	0.5000000000000000	0.1312872901531726
0.1821293208376074	0.3333341358535720	0.2039934389466067
0.2517795397783552	0.5000000000000000	0.2977852343292786
0.3197172996662078	0.3333231638695492	0.3936279493079209
0.3999902224941274	0.5000000000000000	0.4734671812018814
0.4978661908941749	0.3333238007171216	0.5111440636959084
0.5979716357352713	0.5000000000000000	0.4865893569896960
0.6821353026714421	0.3333442358990593	0.4138516083795728
0.7517727010631688	0.5000000000000000	0.3200462231038312
0.8197319433788328	0.3333475046244483	0.2242460292323895
0.9000083067738375	0.5000000000000000	0.1444146986074047
0.9978606521275569	0.6666731255486744	0.1067581080156540
0.0979584996892967	0.8333455961556557	0.1312798111277074
0.1821293208376074	0.6666658931464270	0.2039934389466067
0.2517834751752839	0.8333365721326160	0.2977911339937778
0.3197172996662078	0.6666768651304497	0.3936279493079209
0.3999967118425269	0.8333314590038198	0.4734861465755246
0.4978661908941749	0.6666762282828771	0.5111440636959084
0.5979761406057371	0.8333213150095220	0.4865845014338975
0.6821353026714421	0.6666557931009396	0.4138516083795728
0.7517651600991582	0.8333345096707565	0.3200390197325302
0.8197319433788328	0.6666525243755504	0.2242460292323895
0.9000077069439324	0.8333314595172591	0.1444116860188132
0.0547303646222511	-0.0000000000000000	0.1936920036322874
0.9770518173633880	0.1666818877689538	0.1889688337881588
0.0759762688623124	-0.0000000000000000	0.0437105592349189
0.9516887463402668	0.1666705232777920	0.0404804661059526
0.1908978680610648	-0.0000000000000000	0.3242769502419676
0.1262164848115473	0.1666890252773207	0.2440382299504178
0.2679169005676218	-0.0000000000000000	0.2084165505261288

0.1848530309206524	0.1666840439825450	0.1113873589255364
0.3412929972229500	-0.0000000000000000	0.5143626852716777
0.2578160745596841	0.1666776253648369	0.4199884685148050
0.4028258561107491	-0.0000000000000000	0.3846097093251005
0.3358248996158938	0.1666815099602056	0.3055192231413832
0.5760063052516600	-0.0000000000000000	0.5740915543486061
0.4516796332586341	0.1666930865812364	0.5774170887385430
0.5547326680229341	-0.0000000000000000	0.4241864032740578
0.4770489168077464	0.1666958925384959	0.4289361146876763
0.7679084284008088	-0.0000000000000000	0.4094208773624238
0.6848774698687453	0.1666721776074502	0.5064727846603997
0.6908614724683697	-0.0000000000000000	0.2935381434619490
0.6262229790995270	0.1666792663907662	0.3738393776745256
0.9028184939588542	-0.0000000000000000	0.2332977230599753
0.8358078403866391	0.1666932067492769	0.3123396492495232
0.8413276209756476	-0.0000000000000000	0.1035195257337454
0.7578323363570737	0.1667020968833189	0.1978496526379368
0.0547338167108241	0.3332954814447455	0.1937004533017918
0.9770563649990459	0.5000000000000000	0.1889728760891060
0.0759719186795954	0.3333026664417615	0.0437498789796921
0.9516984556906791	0.5000000000000000	0.0404829781058038
0.1908965093962914	0.3333028275693919	0.3242927023622082
0.1262184250159932	0.5000000000000000	0.2440358670294031
0.2679240607491435	0.3333057358158389	0.2083949191725979
0.1848401381902839	0.5000000000000000	0.1113628452182206
0.3413005615932118	0.3333104470577229	0.5143683418144762
0.2578156498293281	0.5000000000000000	0.4199827348347722
0.4028208854151832	0.3333024935064008	0.3845979074145543
0.3358171714884447	0.5000000000000000	0.3055379645547419
0.5760046754573422	0.3333127488065919	0.5741339748751662
0.4516731545601946	0.5000000000000000	0.5773695663129468
0.5547359827217564	0.3333259016512578	0.4241843959863975
0.4770501418774066	0.5000000000000000	0.4289415950198988
0.7679220232530491	0.3333013171260760	0.4094263888107703
0.6848739573626007	0.5000000000000000	0.5064673734417154
0.6908795465643940	0.3333378597721898	0.2935444741512730
0.6262099034203920	0.5000000000000000	0.3738303399643265
0.9028387368271265	0.3333136554457174	0.2332965585096417
0.8358143203993520	0.5000000000000000	0.3123628600010392
0.8413168922276448	0.3333286130402351	0.1035135734440689
0.7578402422037045	0.5000000000000000	0.1978513680932495
0.0547338167108241	0.6667045475552534	0.1937004533017918
0.9770518173633880	0.8333180832310473	0.1889688337881588
0.0759719186795954	0.6666973625582445	0.0437498789796921
0.9516887463402668	0.8333294477222092	0.0404804661059526
0.1908965093962914	0.6666972014306071	0.3242927023622082
0.1262164848115473	0.8333109457226805	0.2440382299504178
0.2679240607491435	0.6666942931841527	0.2083949191725979
0.1848530309206524	0.8333159270174562	0.1113873589255364

0.3413005615932118	0.6666895819422760	0.5143683418144762
0.2578160745596841	0.8333223456351644	0.4199884685148050
0.4028208854151832	0.6666975354935980	0.3845979074145543
0.3358248996158938	0.8333184610397956	0.3055192231413832
0.5760046754573422	0.6666872801934071	0.5741339748751662
0.4516796332586341	0.8333068844187648	0.5774170887385430
0.5547359827217564	0.6666741273487480	0.4241843959863975
0.4770489168077464	0.8333040784615052	0.4289361146876763
0.7679220232530491	0.6666987118739229	0.4094263888107703
0.6848774698687453	0.8333277933925509	0.5064727846603997
0.6908795465643940	0.6666621692278092	0.2935444741512730
0.6262229790995270	0.8333207046092350	0.3738393776745256
0.9028387368271265	0.6666863735542813	0.2332965585096417
0.8358078403866391	0.8333067642507243	0.3123396492495232
0.8413168922276448	0.6666714159597638	0.1035135734440689
0.7578323363570737	0.8332978741166822	0.1978496526379368
